# Sensitive approach and future perspectives in microscopic patterns of mycorrhizal roots

**DOI:** 10.1038/s41598-019-46743-2

**Published:** 2019-07-15

**Authors:** Vlad Stoian, Roxana Vidican, Ioana Crişan, Carmen Puia, Mignon Şandor, Valentina A. Stoian, Florin Păcurar, Ioana Vaida

**Affiliations:** 1 University of Agricultural Sciences and Veterinary Medicine Cluj-Napoca, Faculty of Agriculture, Department of Microbiology, Cluj-Napoca, 400372 Romania; 2 University of Agricultural Sciences and Veterinary Medicine Cluj-Napoca, Faculty of Agriculture, Department of Plant pathology, Cluj-Napoca, 400372 Romania; 3 University of Agricultural Sciences and Veterinary Medicine Cluj-Napoca, Faculty of Agriculture, Department of Soil ecology, Cluj-Napoca, 400372 Romania; 4 University of Agricultural Sciences and Veterinary Medicine Cluj-Napoca, Faculty of Agriculture, Department of Grasslands and forage crops, Cluj-Napoca, 400372 Romania

**Keywords:** Arbuscular mycorrhiza, Numerical simulations

## Abstract

The harmonization of methodologies for the assessment of radicular endophytic colonization is a current necessity, especially for the arbuscular mycorrhizas. The functionality of mycorrhizal symbionts for plants can be described only by indicators obtained based on microscopic analysis. That is the reason for which a unifying methodology will lead to the achievement of highly correlated indicators comparable from one research to another. Our proposed methodology can further digitize the microscopic observations of colonization. The MycoPatt system is developed as a methodological framework for obtaining objective and comparable microscopic observations. The horizontal, vertical and transversal indicators are highly adaptable and allow the tracking of mycorrhizal colonization in root length. All structures developed by symbionts can be traced and the obtained metadata can be compared without any transformation. Mycorrhizal maps have a high degree of applicability in evaluating soil inoculum efficiency. In the future, the application of this method will lead to digital maps with a high degree of accuracy. MycoPatt allows the mathematical expression of colonization patterns, being a complex model that converts biological data into statistically comparable indicators. This will further allow obtaining inferences with applicative importance and similarity spectra for the colonizing fungi and host plants.

## Introduction

Arbuscular mycorrhizal (AM) fungi belong to phylum Glomeromycota^1,2^ and act as symbiotic connectors for the roots of most land plants in terrestrial ecosystems^3^. They are a group of root obligate symbionts^4,5^ and their role is closely related to plant’s vegetative requirements, from nutrient supplementation to protection against disease, pests and climate stress^6,7^. Mycelium expansion and intraradical growth of AM depends on both host and fungal symbiont species, which is further influenced by environmental conditions^8–11^. This is visible in different root colonization patterns which scores the dependence of host to fungal symbiont^12–14^. The reduced specificity of the AM^15,16^ has allowed the collection of species and their assemblage in bioproducts, largely applicable in agriculture and horticulture. Mycorrhizal symbiotic systems have raised the interest of researchers since their discovery, the research path being separated into three distinct phases: *i)* the phase of exploration in root systems with the identification of main morphotypes and structures; *ii)* an intermediate phase of colonization assessment, specific to applied research in agroecosystems and *iii)* the phase of genetic exploration. *i)*. The temporal analysis starts with Frank, Janse and Gallaud’s incipient research on fungus-root symbiosis^17,18^. The presence of a certain type of mycorrhizal structure was highlighted in the roots of plants. The result was a classification of these symbionts based on their location within root cortex and the intracellular/intercellular development pathway. *ii)*. Since the mid-twentieth century, a number of methods have been proposed to assess the presence of hyphae, certain structures or the level of colonization in root cortex. It was a period with many researchers involved in understanding of nutrient uptake and effects on plant growth^19^. *iii)* After 1990s–2000s, the development of genetic analysis techniques has allowed the complete assessment of symbiotic diversity even for a complete ecosystem resulting in the creation of complex taxonomic databases^20–23^. Etymology of the word mycorrhiza originated from “myco” referring to fungi and “rhiza” referring to root^24^, implies that this associations involves Cormophyta and fungi. Mycorrhizal associations can be classified according to the particular way in which fungi interact with the plant with special reference to distinctive structures formed at symbiotic interface and five main types are generally recognized: Arbuscular mycorrhiza, Ectomycorrhizas, Orchid mycorrhizas, Ericoid mycorrhizas and finally, sub-epidermal mycorrhiza described solely in plant genus *Thysanotus*^25^. First one involves fungi from phylum Glomeromycota, the next three types are formed mainly with Ascomycetes and Basidiomycetes^26^ but in addition, other taxonomic groups such as *Endogone* from Mucoromycotina (Zygomycota) are now known also for establishing plant-root association such as ectomycorrhiza^27^. In the fourth type restricted to genus *Thysanotus*, fungal symbiont was not identified yet^26^. Based on the profile created by Brundrett and Tedersoo^26^ on vascular plants, the highest percentage form AM associations (71%) and within this group the flowering plants represent 72%. However, the methods for the assessment of partnership with AM still have a high degree of subjectivity. From a functional perspective in microscopic techniques, the number and size of root segments, the selection of segments from different architectural areas of the roots, as well as the method of analysis, can lead to very different results even in plants grown under similar conditions. Number of AM fungal species identified following sequencing approaches (including genetically and distinct “cryptic species”) overwhelmingly exceed the species described based on morphometric characteristics^9^. Thus, currently it is difficult to relate molecular data with actual levels of AM diversity identified following species descriptions based on isolated spores from soil, mainly due to limits in distinctive features, and in this regard colonization patterns should be further researched as additional characteristic able to fundament a genetically-identified difference. Soil inoculation with monospecific bioproducts or mycorrhizal consortia is usually assessed from the perspective of increasing the overall level of colonization without taking into account the traceability of species and the development of standard structures by inoculated species^28–30^. From this point of view, it is difficult to calculate the percentage of inoculum participation in the colonization of root system and its impact on the growth and development of hosts. Currently, in the study of AM, methodologies are divided from the beginning into two distinct categories. The first category focuses strictly on the early stages of colonization. They are based on specific methodologies with indicators such as: the number of individual fungal structures created by single root entries (IUM - infection-unit method) and mean infection percentage (MIP), available in Ohtomo *et al*.^31^, or entry points per root cm (EP/cm), as used by Xie and Wu^32^. A second category focuses on advanced colonization stages, with different sets of indicators specific to each method. But, there is a lack of correspondence between these indicators. When tracking temporal development of colonization, the researcher is forced to choose a certain method and then adapt it, which often reduces or makes harder the possibility of comparing it with similar studies^33–36^. However, since the root can be colonized by a series of root endophytic symbionts^37–40^, the lack of a versatile and flexible method is an obstacle in performing high complexity analysis. To better define the consequences for the plant, the methodology should allow a layered mapping of the structures observed and their overlapping for relative inferences at root-level interactions between distinct taxonomic categories of endophytes. Another aspect represents the reduced dissimilarity between developmental patterns of the same AM species in relation to different hosts. The ability to maintain specific structures, or the development of transient or even new structures is not sufficiently explored. And, above all, there is no unified methodology applicable to the whole AM life cycle, both for incipient and advanced stages, which leads to a reduced correspondence between the current obtained indicators. In this paper we propose an improved methodology with high objectivity for the evaluation of AM symbionts based on colonization patterns. The development and mycorrhizal structures in longitudinal sections of root could be explored. Data collection is performed in a matrix system, with observations reported for root segments, separated on microscopic fields. The result is a map of mycorrhizal architecture inside roots. It highlights the structures developed by fungi as well as their expansion in the length and depth of the root.

## Results and Discussion

### Drawbacks in current mycorrhizal assessment

The evaluation of AM mycelium extension in root cortex is essential in establishing the symbiotic status of plants. In current researches mostly 3 methods are used Table 1 – percentage calculation method, grid-line intersect method and magnified intersection method – for each of them the post-processing of collected data and observations being required^41^. Another useful system for rapid evaluation is the root segment estimation proposed by Trouvelot^42^, but the classes of assessment of mycorrhizal indicators are not sufficiently sensitive to variations of colonization between 5–10%. This method is suitable for advanced colonization, with an active exchange of nutrients, but cannot assess early stages of colonization. Analysis is performed on 1 cm long segments and it is recommended to be conducted for a number of 30 or more segments. An addition to the Trouvelot method is the volumetric expression of colonization defined as degree of colonization^43^, determined as a product between frequency and intensity. However, for a real degree of colonization, it is necessary to redefine the manner of assessing the colonization frequency in a similar way as to the intensity. At present, the main difficulty is that none of these methodologies allows the evaluation of all AM structures: entry points (penetration of root cortex) intraradicular mycelium, arbuscules, vesicles or spores. Colonization is assessed as the extent of mycelial expansion and relative to the whole root aggregate without providing information on development patterns. AM researches follow two main directions^44–47^: 1) symbiotic status of some species focusing on the potential for installation/persistence of inoculum in the rhizosphere or 2) soil-plant-mycorrhizal interaction. Current methodologies provide general information on the presence of symbiotic fungi in the root, lacking the overall picture of the hyphae path in the root and the structure’s development pattern. Another drawback is that each methodology leads only to a low number of indicators, in some cases just one, and often there is no direct correspondence between them across different methods. This forces the researcher from the beginning to express the results of observations in a form that limits the comparability with the results of other researches based on other methodologies. Particularities of spores and germlings, characteristics to developed structures are described only for trap cultures in AM specialized collections^48^. An additional character is the morphological class to which they belong, based on the type of arbuscules development: the Arum type, defined by intercellular hyphae and intracellular branched-arbuscules, and Paris type with coiled intracellular hyphae and arbuscules^49^. However, the AM inoculum to different species, in field and pots, reveals the potential for appearance of different structures during the colonization process^28,50–52^. Also there is the possibility of lack of sporulation (absence or deficiency) in pots for some species. There are transient characters of arbuscules - models between *Arum* and *Paris*, a wide range of branching patterns of the mycelium for different root types, also the development of irregular forms of vesicles. Deguchi *et al*.^53^ proposes the scanning of the entire root with the WinRHIZO software as a quick estimation method of AM colonization. This adaptation of the modified grid-line intersection method leads to a linear evaluation of the root-fungus system and allows realistic results to be obtained over for entire colonization. The drawback of the method is that it does not effectively assess the location of each type of mycorrhizal structure and its ratio to the colonized surface, necessary in order to identify the array pattern and repeatability. The mechanism is developed as the integrative potential of mycorrhizas with root systems, for the analysis of the nutrient flows between the symbionts, the separation of colonization according to root architecture and the collection of datasets regarding the development strategies of both partners^54^. Öpik and Davison^55^ highlight the need for DNA sequencing of existing AM collections for a common marker region. Likewise, it is necessary to correlate mycorrhizal genomics studies with plant traits, colonization mechanism, evaluation of the reaction between partners and the presence of symbiosis in assemblage of communities. So far, there are collections of mycorrhizal spores, a wide variety of monospecific or consortia bioproducts, but there is no objective methodology to track the inoculum in the applied plants and the differentiation from native symbiotic flora. Until now, researches into AM mechanisms have produced a high number of results^56–58^, but with sparse cohesion. The large number of existing researches and databases present a difficulty for comparisons for researchers in order to establish the level of scientific applicability in the field. This makes it virtually impossible to extrapolate realistic conclusions even by interpolating a large number of metadata.

**Table 1:** Synthesis of AM microscopic evaluation methods.

Assessment of:	Structures*					Indicators		References
***Root segments***	EP	H	A	V	S	1	>1	
mycorrhizal segments		x	x			x		Daft *and* Nicolson^61^
grid-line intersect		x	x			x		Giovannetti *and* Mosse^62^
intensity estimation		x	x			x		Trouvelot *et al*.^42^
**Assessment of:**	Structures*					**Indicators**		
***Microscopic Field of view***	EP	H	A	V	S	1	>1	
mycorrhizal fields of view		x	x			x		Baylis^63^
magnified intersections		x	x	x			x	McGonigle P. T. *et al*.^64^

Note:structures specifically mentioned by authors in their methodology, where – EP = entry point, H = hyphae, A = arbuscules, V = vesicles, S = spores.

### Testing the performance of MycoPatt

In order test the performance of MycoPatt model, 4 species grown in similar open field conditions were selected: *Allium porrum*, *Plantago major*, *Trifolium repens* and *Zea mays*. Prior scoring the mycorrhizal status all roots were cleared in a solution of NaOH (10%) and stained with blue ink – Pelikan (5%) and vinegar (5%), according to a modified ink-vinegar method^59^. Our analysis system responded well to the scoring of colonization at a reduced scale. We obtained an increase in the sensitivity of observations simultaneously with the collection of great amount of data (Supplementary Datasets 1–4) that provide realistic average values of the colonization mechanism (Table 2). Indicators obtained as well as colonization map (Supplementary Fig. 1) indicates differences between AM patterns of development and expansion inside root, among tested species. Mean values for frequency and intensity of colonization are comparable within the interval ±10%. Analysis of the mycorrhizal maps reveals also a pattern of alternation among mycorrhizal hot-spots and mycorrhizal-free areas. Arbuscules abundance is similar for *Plantago major* and *Zea mays*, but arbuscules distribution within root is more uniformly in *Zea mays*. By contrast, *Allium porrum* and *Trifolium repens* register large differences in value for arbuscules/vesicles as well as their distribution along root length. Observations revealed 1% entry of points inside root, which corresponds with a predominately intraradicular development. Ratio between mycorrhizal and non-mycorrhizal areas is just 0.30 in *Allium porrum* and reaches a maximum in species *Trifolium repens*, which indicate that the development of a large number of AM structures inside the root in this case.

**Table 2:** Comparative analysis of mycorrhizal patters in 4 species.

Parameter	*Allium porrum*		*Plantago major*		*Trifolium repens*		*Zea mays*	
	Mean	SE	Mean	SE	Mean	SE	Mean	SE
F	52.53	5.19	62.20	3.58	60.27	5.20	64.00	4.20
I	21.93	1.81	30.53	2.19	32.27	4.26	28.87	2.67
A	2.60	1.16	5.53	1.76	8.60	2.30	5.40	1.74
V	0.13	0.13	1.53	0.93	1.27	0.46	2.33	0.84
S			2.73	1.19	0.53	0.41		
EP	0.80	0.33	1.13	0.27	0.60	0.21	0.67	0.27
nonM	75.07	1.94	64.40	2.31	61.60	5.35	65.07	3.07
CD	12.42	1.62	19.63	2.08	22.19	4.14	19.74	2.69
M/nonM	0.30	0.03	0.50	0.05	0.76	0.22	0.49	0.06

### Future prospects and insertion of MycoPatt in mycorrhizology

**Figure 1:**
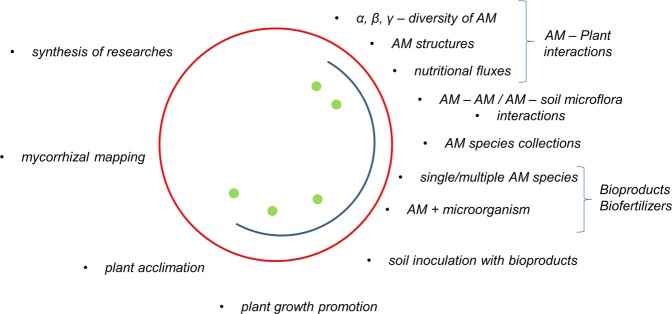
Insertion levels of MycoPatt model in mycorrhizology. Researchers (red circle); Producers (blue semicircle); Agronomists (green dots).

Model MycoPatt reassures an objective evaluation of AM level in radicular cortex, allowing the change of observation angle through the use of grids from the microscopic eye-piece (10 × 10) or the analysis system of microscopic images. Thus, maps of colonization at different levels of resolution and their exact reporting of these to the root length can be created. These aspects support the performance of the model, which adds to the microscopy a high resolution of the observations, the speed of analysis and the fidelity of the produced data, which promotes it as an innovative technique in the field. Like previous methods, MycoPatt assesses the endophytic stage of colonization, thus taking into account vesicles, arbuscules and intraradicular hyphae and spores, that develop inside the root. The exception are the auxiliary cells which although form at the exterior of the root they are not present in all AM fungi taxa and thus might be worth noticed in some cases. Compared with the existing techniques the methodology that we propose reduces subjectivity in the analysis of AM symbionsis. Currently, the roots are evaluated either by reference to a grid system of different sizes, followed by quantification of observed intersections or by cutting into 1 cm segments, and then analyzed randomly under a microscope. The first system produces an evaluation of the presence of mycorrhizae, but without detailing the structures inside the root cortex. The second system uses a different number of root segments, and the evaluation refers to an entire segment regardless of the objective used for microscopy. This methodology is based on personal bias in assessment the level of colonization. MycoPatt evaluates in detail how the AM is expanded, each indicator being calculated separately horizontally and vertically for each microscopic field. In this way, final data report is objectively made and present the real level of structures extension in roots. Another result is the achievement of a clear separation between the mycorrhized and non-mycorrhized areas. Up to date, it is the only method that allows the evaluation of all AM structures at once and their real position in roots. The system is applicable for samples collected in the open field either for study of colonization in different phenophase or as a reaction to experimental variables. For plants grown in pots, where usually the entire root is sampled the system allows the efficient monitoring of AM colonization development and the correlation of colonization pattern with the root architecture. One of the major advantages of MycoPatt insertion in mycorrhizology is the ability to locate the exact and real position of structures on the map of radicular cortex. Harmonization of mycorrhiza studies based on the MycoPatt methodology will lead to the creation of a unitary system of data collection, and it will increase the flexibility of researches and applications. Numeric expression of colonization patterns will open a new stage in mycorrhizology, togheter with the creation of new sets of indicators adapted for a realistic description of symbiotic mechanisms. New orientation for these could be towards the whole AM mechanism or only for the assessment of presence/absence of the structures of interest for the researcher, producers of bioproducts or agronomists, as end-uses for this information in the field (Fig. 1):

- For researchers, the utility of MycoPatt resides in the unifying character of the model that will allow high-precision comparations without a conversion of data. Assembling the mycorrhizal data collections will facilitate the identification of stress factors over the development of symbiosis. The model folds on the holistic approach in regards with functionality of AM development in ecosystems and under experimental conditions. From a similar perspective it is necessary also a mapping of host plant colonization by AM from current collections, activity for which MycoPatt presents a high level of adaptation. Further, the researches could focus either on describing mycorrhizal inoculum in relation with host plants or for identification of colonization patterns in native flora. The new context will allow the isolation of new species and strains that will complete the digitization system of colonization patterns. In phytosociology studies a mycorrhizal mapping is necessary for establishing the biologic bases for trans-rhizosphere inter-relationships of plants from ecosystems in order to understand nutritional climax, occurrence and establishment of invasive species, perennity of phytocoenosys and prediction of future successions.
- Bioproducts producers will benefit from an efficient analysis system of selected species for creation of inoculum and MycoPatt will allow the assessment of performance of each AM species selected in relation with tester plants. Concomitantly, the analysis capacity of interactions between AM-plants-other microbial groups and directing the selection towards obtaining synergistic effects will grow. Synthesis of bioproducts will evolve towards high efficiency inoculum (mono and pluri-specific) assembled according with certain plants/ecosystem, through a fast and sensitive testing method.
- In agronomy and plant breeding, the model is performant for precise evaluation of adaptability potential of a given species or variety in different soil conditions with regards to symbiotic soil flora responsible for nutritional networks. Relatively to soil it can be used to evaluate the native mycorrhization potential, capacity of supporting the plant-grow promoting phenomena and temporal stability. Another direction is the evaluation of survival and efficiency of inoculum (quality of bioproducts) for different species.

Flexibility of the proposed methodology allows the conversion towards symbiotic root endophyte evaluation studies (dark septate endophytes, fine root endophytes), or the inclusion of root pathogens. From some studies the interest for comparison of colonization by different endophyte types is evident, a fact that determined the adaptation of some mycorrhizology methods^60^. From an ecologic and biologic point of view, the model constitutes an important segment in the research of mechanisms related to interactions between roots and fungi, as well as identification of symbiotic or pathogen potential. MycoPatt is the methodologic framework of a prototype for digital analysis of microscopic investigations, with wide range of application and universal indicators. Integration of observations in centralized databases will allow comparisons that could increase applicative value of results. In this way, the amount of data available for future researches increases, leading to higher potential for extracting punctual comparisons and conclusions. Based on obtained observations new mycorrhizal indicators could be proposed, such as models for histologic gradients of discontinuity for root endosymbionts, especially redefining, with more precise terms, the dependence status of plants for symbionts. With other words, the system will represent a starting point in redefining the role of microscopy in current research on root endophytes. Implementation of the described methodologic prototype will improve data management of results from researches, once with the potential growth for implementation and interpolation of metadata. Mycorrhizal digital maps will have a high level of cohesion. The results obtained through one methodology will allow comparison of a high number of researches. Such a future application will allow instant comparations and automatic overlapping through the use of a complex set of indicators. Evolution of this methodology will lead to digitization of AM systems with one unified system of indicators. Also, it will allow the creation of databases which will come to supplement the existing ones for molecular taxonomy of AM species or those dedicated to descriptive characteristics of spores.

## Conclusions

MycoPatt model is an objective system ready to use and less time consuming. Complexity of the system derives from the harmonization of pre-existing evaluation methodologies used in assessing efficiency of mycorrhizal colonization. High resolution of MycoPatt allows the creation of realistic databases over unique AM colonization patterns in root cortex. The indicators set is integrated in a model that allows the expression of mycorrhizal mechanisms in a numeric form, easy to digitalize and to analyse statistically. Flexibility and adaptive capacity allow the extension of MycoPatt to all radicular or pathogenic endophytes. Future perspectives of the system are to normalize and to increase the efficiency of information flow between researchers and those who implement mycorrhizological applications in field.

## Method

### Concepts of mycorrhizal patterns method

**Figure 2:**
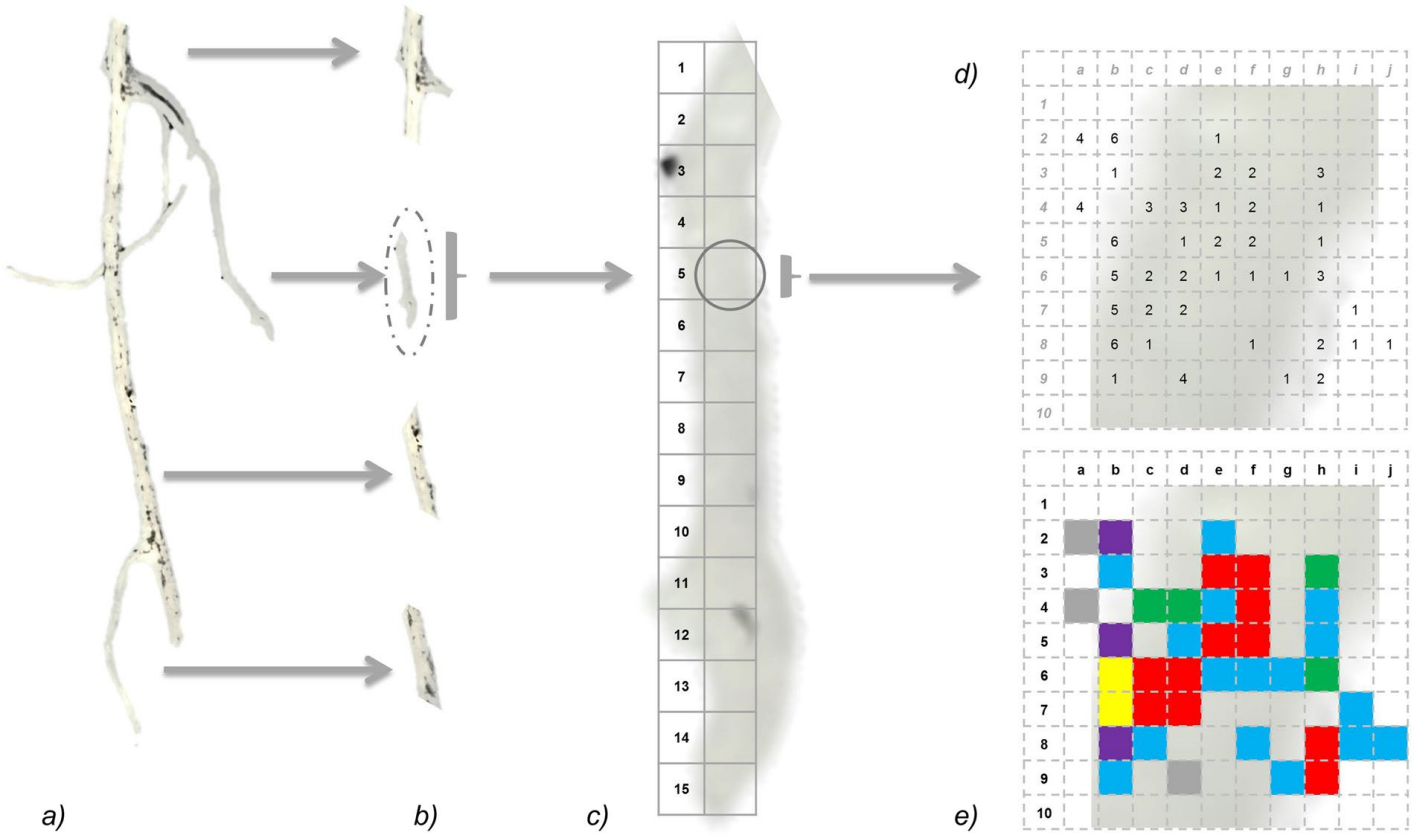
Schematic framework of MycoPatt model. (**a**) Root system; (**b**) selection of 1 cm segments; (**c**) segment analysis flow - 15 microscopic fields/segment; (**d**) MycoPatt grid overlay on each microscopic field with the assignment of values for mycorrhizal structures: (1) hyphae, (2) arbuscules, (3) vesicles, (4) spores, (5) auxiliary cells, (6) entry points; (**e**) conversion of values into colors for development of mycorrhizal map: (1) blue, (2) red, (3) green, (4) gray, (5) yellow, (6) purple.

The MycoPatt model proposed in this paper for assessing the mycorrhization takes into account the linear segmentation of root samples in 1 cm segments and the separate evaluation on each microscopic field of AM structures (Fig. 2a,b). The size of root fragments for microscopic evaluation corresponds to a number of 15 microscopic fields at 400× (Fig. 2b,c). According to the legend, each structure is encoded in the data collection table (Supplementary Table 1) in a matrix of 10 × 10 squares (Fig. 2d). The evaluation and coding can be done directly at the microscope by using the Eyepiece micrometer grid (10 × 10) or by applying 10 × 10 grids to images captured on the microscope. In this way it is possible to follow the entrance path for hyphae in the root and the development of mycelium in horizontal and vertical plans. Observations can be made about the penetration of hyphae into intracellular spaces and occurrence of arbuscules, vesicles, intraradical spores and auxiliary cells. The model is adapted also for thicker roots and can be used without any changes at different microscopic magnification (e.g. 100×, 200×). Collected data is transferred to an Excel data table (Supplementary Dataset 5), which will automatically convert each microscopic field into a mycorrhizal map (Fig. 2e). The workbook presented in Appendix 2 is separated into 3 sheets, the first (*rawdata*), the database – contains the values of the microscopic observations; the second (*graphs*) elaborates the mycorrhizal map based on the color codes and the third (*parameters*) is designed for assemblage of numerical values of mycorrhizal parameters. Finally, a colored map of each 1 cm segment is obtained and can be assembled into a complex model following the pattern of mycorrhizal development throughout the root system. The advantage of MycoPatt is that it tracks the establishment and development of symbiosis with AM fungi along root length, highlighting the entry areas in the cortex and branches. It is possible to correlate data obtained with root architecture and plant dependence to mycorrhizas. The background formulas are adaptable and the coding can be changed with a minimal effort depending on the user’s needs. The colored map obtained is developed for AM and represents the expression of microscopic evaluation. If needed it can be adapted for dark septate or fine root endophytes.

### Redefining the colonization parameters

Analysis of AM fungi development in root system based on the MycoPatt model imposed a redefinition of mycorrhizal parameters. Indicators are divided into three categories for a realistic assessment of mycelium extension and the pattern of mycorrhizal structures (Table 3). Category *h* - *horizontal* parameters, calculated separately for each line of the microscopic field; *v* - *vertical* parameters, calculated separately for each column of the microscopic field; *t* - *transversal* parameters, calculated for the entire surface of the microscopic field (Fig. 2d). The final report of all values obtained across the entire root is calculated as the mean of the transversal parameters. Mycorrhizal frequency is the relative value of the colonization incidence in analyzed units, which separates, on both horizontally and vertically plans, the areas that have been mycorrhized by the non-mycorrhized ones. Compared to previous methodologies, colonization frequency is calculated on a scale from 0–100%, with 10% divisions corresponding to each line or column of the microscopic field. Horizontal frequency (Fh) sums all rows from a microscopic field where AM structures are detected and asses to each line the value of 10%. Similarly, vertical frequency (Fv) sums all columns positive for AM structures. Due to 10% scale used for this parameter, both Fh and Fv can be overlapped and the obtained transversal frequency (Ft) normalizes the results. Colonization intensity represents the value of mycorrhizal expansion in analyzed units. The parameter is calculated as the sum of squares, on rows or column, where AM structures are detected. It is also extracted separately on each line and column, each square representing a value of 1%. Thus, the scale of appreciation is 0–100%, but with 1% divisions, which makes it easier to track AM development in root. Similarly to intensity, the presence and abundance of arbuscules, vesicles, spores and auxiliary cells is evaluated. The MycoPatt model allows tracing these structures in their true position in the root cortex. The large resolution of the model allows longitudinal root analysis, segmentation based on root architecture and redefinition of the microscopic analysis of the entire general symbiotic mechanism. An important point is the evaluation of entry points. In this way a reference can be done to the intraradicular development of the entire fungal assembly. Frequency and intensity synthesis is expressed as the degree of colonization and gives the perspective of the total volume explored by AM in root. This parameter is calculated only at the transversal level, and requires the data obtained both horizontally and vertically. To determine the ratio of mycorrhized/non-mycorrhized areas the number of colonization-free areas is also calculated.

**Table 3:** Assessment of AM parameters in root system.

Parameter	Code	Unit	Horizontal	Vertical	Transversal	Root assesment
			*h*	*v*	*t*	Average of *t*
Frequency	F	%	∑ F_a-j_	∑ F_1-10_	(∑ Fh $\ast$ ∑ Fv)/100	Av (Ft)
Intensity	I	%	∑ I_a-j_	∑ I_1-10_	∑ I	Av (It_1-15_)
Arbuscules	A	%	∑ A_a-j_	∑ A_1-10_	∑ A	Av (At_1-15_)
Vesicles	V	%	∑ V_a-j_	∑ V_1-10_	∑ V	Av (Vt_1-15_)
Spores	S	%	∑ S_a-j_	∑ S_1-10_	∑ S	Av (St_1-15_)
Aux cells	AX	%	∑ AX_a-j_	∑ AX_1-10_	∑ AX	Av (AXt_1-15_)
Entry points	EP	%	∑ EP_a-j_	∑ EP_1-10_	∑ EP	Av (EPt_1-15_)
Non-mycorrhized areas	nonM		∑ nonM_a-j_	∑ nonM_1-10_	∑ nonM	Av (nonMt_1-15_)
Colonisation degree	CD	%			(Ft $\ast$ It)/100	Av (CDt_1-15_)
Myco/nonMyco report	M/nonM				It/nonMt	Av (M/nonMt_1-15_)

## Supplementary information

Supplementary_Dataset_1

Supplementary_Dataset_2

Supplementary_Dataset_3

Supplementary_Dataset_4

Supplementary_Dataset_5

Supplementary Information
