# Supplementary material for: Sensitive approach and future perspectives in microscopic patterns of mycorrhizal roots
Source: Sci Rep. 2019 Jul 15;9:10233. doi: 10.1038/s41598-019-46743-2 (PMC6629619; doi:10.1038/s41598-019-46743-2)
Supplement: Supplementary file 6 — Supplementary Information [file 41598_2019_46743_MOESM6_ESM.pdf]

Sensitive approach and future perspectives in microscopic patterns of mycorrhizal roots

Vlad Stoian[1]*, Roxana Vidican[1]*, Ioana Crișan[1], Carmen Puia[2], Mignon Șandor[3], Valentina A. Stoian[3], Păcurar Florin[4] and Ioana Vaida[4]

[1]University of Agricultural Sciences and Veterinary Medicine Cluj-Napoca, Faculty of Agriculture, Department of Microbiology, Cluj-Napoca, 400372, Romania

[2]University of Agricultural Sciences and Veterinary Medicine Cluj-Napoca, Faculty of Agriculture, Department of Plant pathology, Cluj-Napoca, 400372, Romania

[3]University of Agricultural Sciences and Veterinary Medicine Cluj-Napoca, Faculty of Agriculture, Department of Soil ecology, Cluj-Napoca, 400372, Romania

[4]University of Agricultural Sciences and Veterinary Medicine Cluj-Napoca, Faculty of Agriculture, Department of Grasslands and forage crops, Cluj-Napoca, 400372, Romania

**corresponding authors:* Dr. Vlad Stoian, University of Agricultural Sciences and Veterinary Medicine Cluj-Napoca, Faculty of Agriculture, Department of Microbiology, Cluj-Napoca, Calea Mănăștur st., no 3-5, 400372, Romania. vlad.stoian@usamvcluj.ro

Prof. Roxana Vidican, University of Agricultural Sciences and Veterinary Medicine Cluj-Napoca, Faculty of Agriculture, Department of Microbiology, Cluj-Napoca, Calea Mănăștur st., no 3-5, 400372, Romania. roxana.vidican@usamvcluj.ro

Supplementary Table 1. Data collection table

		a	b	c	d	e	f	g	h	i	j
1	1										
	2										
	3										
	4										
	5										
	6										
	7										
	8										
	9										
	10										
2	1										
	2										
	3										
	4										
	5										
	6										
	7										
	8										
	9										
	10										
3	1										
	2										
	3										
	4										
	5										
	6										
	7										
	8										
	9										
	10										
4	1										
	2										
	3										
	4										
	5										
	6										
	7										
	8										
	9										
	10										
5	1										
	2										
	3										
	4										
	5										
	6										
	7										
	8										
	9										
	10										
6	1										
	2										
	3										
	4										
	5										
	6										
	7										
	8										
	9										
	10										
7	1										
	2										
	3										
	4										
	5										
	6										
	7										
	8										
	9										
	10										
8	1										
	2										
	3										
	4										
	5										
	6										
	7										
	8										
	9										
	10										
9	1										
	2										
	3										
	4										
	5										
	6										
	7										
	8										
	9										
	10										
10	1										
	2										
	3										
	4										
	5										
	6										
	7										
	8										
	9										
	10										
11	1										
	2										
	3										
	4										
	5										
	6										
	7										
	8										
	9										
	10										
12	1										
	2										
	3										
	4										
	5										
	6										
	7										
	8										
	9										
	10										
13	1										
	2										
	3										
	4										
	5										
	6										
	7										
	8										
	9										
	10										
14	1										
	2										
	3										
	4										
	5										
	6										
	7										
	8										
	9										
	10										
15	1										
	2										
	3										
	4										
	5										
	6										
	7										
	8										
	9										
	10										

Legend

1		Hyphae
2		Arbuscules
3		Vesicles
4		Spores
5		Auxiliary cells
6		Entry points

Supplementary Figure 1.

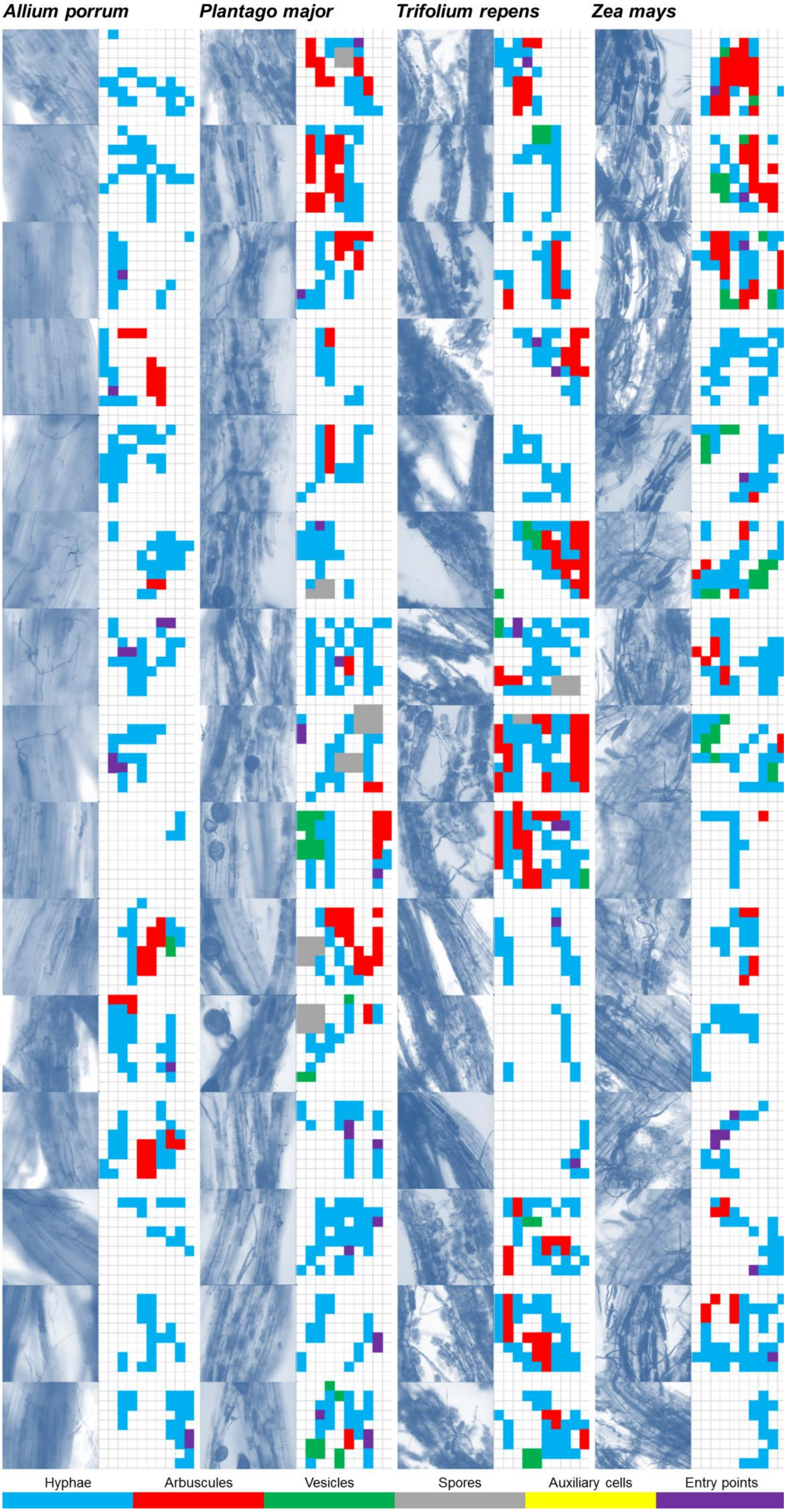

Supplementary Figure 1: AM colonization map of 4 species generated by MycoPatt: *Allium porrum, Plantago major, Trifolium repens* and *Zea mays*
